# Efficient and robust optimization of nuclear and electronic orbitals within the nuclear Hartree product representation

**DOI:** 10.1063/5.0308634

**Published:** 2026-02-21

**Authors:** Mathew Chow, Eno Paenurk, Sharon Hammes-Schiffer

**Affiliations:** 1 Department of Chemistry, Yale University, New Haven, Connecticut 06520, USA; 2 Department of Chemistry, Princeton University, Princeton, New Jersey 08544, USA

## Abstract

The nuclear–electronic orbital (NEO) approach treats electrons and specified nuclei quantum mechanically on the same level, providing a straightforward framework for including nuclear quantum effects beyond the standard Born–Oppenheimer approximation. Both density functional theory and wave function methods have been developed within the NEO framework. Despite its success, some NEO applications are limited by the intrinsically difficult self-consistent field (SCF) convergence of the Hartree–Fock or Kohn–Sham electronic and nuclear orbitals. Herein, we demonstrate that using the nuclear Hartree product representation improves the efficiency and stability of the NEO-SCF optimization procedure compared to the nuclear Slater determinant representation. Both representations lead to the same NEO-SCF energy because the proton–proton exchange energy is typically much smaller than the SCF energy convergence threshold. Faster and more robust NEO-SCF convergence is observed using this strategy for a test set of 92 molecules containing multiple quantum protons. We determine that the improved convergence behavior stems from the absence of nuclear self-Coulomb and self-exchange terms in the Hartree product representation. Furthermore, we show that both electronic and nuclear orbitals can be optimized simultaneously through extensions of the geometric direct minimization and trust-radius augmented Hessian algorithms. These algorithms can be useful for converging challenging systems, as demonstrated by calculations on the enzyme ribonucleotide reductase and the UO_2_(OH)_4_ complex. The findings in this work will enable more efficient and robust NEO calculations for a wide range of applications.

## I. INTRODUCTION

The nuclear–electronic orbital (NEO) framework^1–3^ incorporates nuclear quantum effects and non-Born–Oppenheimer effects into first-principles quantum chemistry methods, namely, *ab initio* wave function approaches and density functional theory (DFT). In the NEO framework, electrons and specified nuclei, usually protons, are treated on equal footing without the standard Born–Oppenheimer separation between them. Both ground state and excited state approaches have been developed within the NEO framework to enable the inclusion of nuclear quantum effects into quantum chemistry calculations and molecular dynamics simulations.^4–12^ This approach is especially useful in chemical and biological systems for which effects such as anharmonicity, zero-point energy, and nuclear delocalization are expected to play an important role.

Similar to their conventional electronic Hartree–Fock or Kohn–Sham counterparts, all NEO methods first require self-consistent field (SCF) convergence of the molecular orbitals to a stationary solution. Solving the NEO-SCF problem can be challenging because it requires convergence of both the electronic and nuclear subsystems, which are strongly coupled to each other through attractive Coulombic interactions as well as mixed-particle correlation terms for NEO-DFT methods. Compared to the conventional electronic SCF procedure, the NEO-SCF procedure often requires many more iterations to reach convergence. To improve computational feasibility, a simultaneous optimization algorithm^13^ based on the direct inversion of iterative subspace (DIIS) algorithm^14,15^ was developed. For systems with many quantum protons, we have shown that the simultaneous DIIS solver can potentially reduce the computational cost compared to a stepwise optimization algorithm, wherein the electronic and nuclear subsystems are each partially or fully converged in alternating fashion. Beyond the various SCF strategies, other techniques for improving computational scaling, such as density-fitting^16–18^ and Cholesky decomposition,^19^ have also been developed.

The fundamental reason for the complexity of the NEO-SCF procedure and the unfavorable number of convergence iterations is currently not well understood. One hypothesis is that the challenging nature of convergence is simply due to the complex interplay between electrons and protons. A second hypothesis proposed by Reiher and co-workers is that the nuclear energy landscape has a higher degree of nonconvexity than the electronic landscape and that interparticle-type coupling is a less prominent factor.^20^

In this work, we propose that the nuclear self-Coulomb and self-exchange interactions could be leading factors behind the additional complexity of NEO-SCF optimizations compared to conventional electronic SCF optimizations. To support this hypothesis, we report a significant speedup in NEO-SCF optimizations through a substantial reduction in the number of convergence iterations when these nuclear self-interactions are not included. These interactions are absent when the distinguishable particle representation^21^ is invoked by using a Hartree product rather than a Slater determinant representation for the quantum nuclei. By construction, the nuclear Hartree product representation neglects all nuclear–nuclear interactions except for the nuclear–nuclear classical Coulomb terms. In most NEO applications, the quantum nuclei are protons, which will be the case herein. Previous work showed that proton–proton exchange and correlation energies are negligible compared to their electronic counterparts.^2,21^

We also demonstrate that the NEO-SCF optimization is often very sensitive to the choice of the initial guess for the molecular orbitals and, as a result, is highly prone to convergence instabilities when a poor guess is used. The problems with the choice of initial guess are eliminated almost entirely by invoking the nuclear Hartree product representation.^21^ We note that Ref. 21 presented the nuclear Hartree product representation for NEO-DFT without electron–proton correlation using a stepwise optimization algorithm. Herein, we present the nuclear Hartree product representation for NEO-DFT, including electron–proton correlation, with more sophisticated simultaneous optimization algorithms. Moreover, our analysis shows that the improved performance arises mainly from the absence of the nuclear self-Coulomb and self-exchange terms in the Hartree product representation. This fundamental understanding will be important for future algorithmic developments.

In addition, we extend the simultaneous NEO-SCF optimization scheme to the geometric direct minimization (GDM)^22^ and trust-radius augmented Hessian (TRAH)^23,24^ solvers to enable optimization of both electronic and nuclear orbitals at the same time using Newton–Raphson techniques. A related multicomponent, simultaneous GDM and TRAH scheme has recently been developed in the context of NEO multiconfigurational self-consistent field orbital optimization.^25^ In addition, another SCF algorithm similar to TRAH has been implemented to enhance the convergence of nuclear–electronic SCF calculations.^20^ Herein, we present the simultaneous GDM algorithm with both the nuclear Slater determinant and nuclear Hartree product representations and the simultaneous TRAH algorithm with the nuclear Slater determinant representation. To the best of our knowledge, these algorithms have not been implemented previously for NEO-DFT. We show that these approaches are generally useful for converging NEO-SCF cases where the DIIS solver fails.

An outline of this paper is as follows: In Sec. II, we review the NEO Hartree–Fock (NEO-HF) approach in the context of nuclear Slater determinant and nuclear Hartree product representations, followed by a brief discussion of the key aspects of NEO-DFT. Then we describe the various simultaneous NEO-SCF algorithms, along with common choices for the initial guess. Section III provides the computational details for the calculations performed in this work, and Sec. IV presents these calculations. Finally, our concluding remarks are provided in Sec. V.

## II. METHODS

### A. NEO Hartree–Fock

In the NEO approach, the system is divided into $N^{\text{e}}$ electrons, $N^{\text{p}}$ quantum protons, and $N^{\text{c}}$ classical nuclei. In atomic units, the NEO Hamiltonian can be written as

(1) $${\overset{ˆ}{H}}_{\text{NEO}}=-\sum_{i}^{N^{\text{e}}}\frac{1}{2}\nabla_{i}^{2}+\sum_{i}^{N^{\text{e}}}\sum_{j>i}^{N^{\text{e}}}\frac{1}{\left|r_{i}-r_{j}\right|}-\sum_{i}^{N^{\text{e}}}\sum_{A}^{N^{\text{c}}}\frac{Z_{A}}{\left|r_{i}-R_{A}\right|}-\sum_{i^{\prime }}^{N^{\text{p}}}\frac{1}{2m_{\text{p}}}\nabla_{i^{\prime }}^{2}+\sum_{i^{\prime }}^{N^{\text{p}}}\sum_{j^{\prime }>i^{\prime }}^{N^{\text{p}}}\frac{1}{\left|r_{i^{\prime }}-r_{j^{\prime }}\right|}+\sum_{i^{\prime }}^{N^{\text{p}}}\sum_{A}^{N^{\text{c}}}\frac{Z_{A}}{\left|r_{i^{\prime }}-R_{A}\right|}-\sum_{i}^{N^{\text{e}}}\sum_{i^{\prime }}^{N^{\text{p}}}\frac{1}{\left|r_{i}-r_{i^{\prime }}\right|}+\sum_{A}^{N^{\text{c}}}\sum_{B>A}^{N^{\text{c}}}\frac{Z_{A}Z_{B}}{\left|R_{A}-R_{B}\right|},$$

where $\{i,j,\ldots \},\left\{i^{\prime },j^{\prime },\ldots \right\}$, and {A,B,…} are the indices for electrons, quantum protons, and classical nuclei, respectively. Here, $m_{\text{p}}$ represents the proton mass, $Z_{A}$ represents the classical nuclear charge, R represents the position of a classical nucleus, and r represents the position of either an electron or a quantum proton, depending on the subscript.

Within the NEO-HF ansatz, the wave function can be expressed as a product of electronic and protonic Slater determinants,

(2) $$\Psi \left(x^{\text{e}},x^{\text{p}}\right)=\Phi^{\text{e}}\left(x^{\text{e}}\right)\Phi^{\text{p}}\left(x^{\text{p}}\right),$$

where $\Phi^{\text{e}}$ and $\Phi^{\text{p}}$ are the determinants consisting of electronic and nuclear spin orbitals, respectively. Typically, the electronic and nuclear spatial orbitals are expanded in Gaussian-type basis functions. Obtaining the NEO-HF energy requires iterative, self-consistent convergence of the set of NEO-HF Roothaan equations,

(3) $$F^{\text{e}}C^{\text{e}}=S^{\text{e}}C^{\text{e}}\varepsilon^{\text{e}},$$

(4) $$F^{\text{p}}C^{\text{p}}=S^{\text{p}}C^{\text{p}}\varepsilon^{\text{p}},$$

where $F^{e},S^{e},C^{e}$, and $\varepsilon^{e}$ are the electronic Fock matrix, overlap matrix, coefficient matrix, and orbital energy matrix, respectively, and the quantum nuclear matrices are defined analogously.

Protons are highly localized in molecular systems, and the proton–proton exchange terms have been shown to be negligible, often eight to ten orders of magnitude smaller than the corresponding electron–electron exchange terms.^2^ As a result, the NEO-HF wave function given in Eq. (2) can be reformulated to treat the quantum protons as distinguishable particles,^21^

(5) $$\Psi \left(x^{\text{e}},x^{\text{p}}\right)=\Phi^{\text{e}}\left(x^{\text{e}}\right)\prod_{i^{\prime }=1}^{N^{\text{p}}}\chi_{i^{\prime }}^{\text{p}}\left(x_{i^{\prime }}^{\text{p}}\right).$$

Here, the nuclear wave function is represented as a Hartree product of $N^{\text{p}}$ quantum protons, where $\chi_{i^{\prime }}^{\text{p}}$ are the nuclear spin orbitals, and the electronic wave function is still represented as a Slater determinant. The nuclear spatial orbital for each distinguishable nucleus is expanded in its own Gaussian-type basis set. In this regime, the full $N^{\text{p}}$-nuclei Roothaan equation [Eq. (4)] is replaced by $N^{\text{p}}$ single-nucleus equations. In other words, the one-particle nuclear Fock operator expressed in the Slater determinant representation as^1^

(6) $$f^{\text{p}}\left(r_{1}^{\text{p}}\right)=h^{\text{p}}\left(r_{1}^{\text{p}}\right)+\sum_{j^{\prime }}^{N^{\text{p}}}\left[J_{j^{\prime }}^{\text{p}}\left(r_{1}^{\text{p}}\right)-K_{j^{\prime }}^{\text{p}}\left(r_{1}^{\text{p}}\right)\right]-2\sum_{i}^{N^{\text{e}}/2}J_{i}^{\text{e}}\left(r_{1}^{\text{p}}\right)$$

is replaced by a different Fock operator for each $i^{\prime }\text{th}$ distinguishable nucleus in the nuclear Hartree product representation,

(7) $$f_{i^{\prime }}^{\text{p}}\left(r_{i^{\prime }}^{\text{p}}\right)=h^{\text{p}}\left(r_{i^{\prime }}^{\text{p}}\right)+\sum_{j^{\prime }\neq i^{\prime }}^{N^{\text{p}}}J_{j^{\prime }}^{\text{p}}\left(r_{i^{\prime }}^{\text{p}}\right)-2\sum_{i}^{N^{\text{e}}/2}J_{i}^{\text{e}}\left(r_{i^{\prime }}^{\text{p}}\right).$$

Here, $h^{\text{p}}$ is the nuclear core Hamiltonian operator, and J and K are the Coulomb and exchange operators, respectively, with superscripts indicating the electrons or protons. These equations are valid for a restricted closed-shell treatment of the electrons, but the extension to an open-shell treatment of the electrons is straightforward. In the nuclear Slater determinant representation, the protons are assumed to be high spin with each spatial protonic orbital singly occupied. In the nuclear Hartree product representation, the protons are distinguishable and have no nuclear spin interactions.

Equation (7) differs from Eq. (6) in that the exchange operator $K^{\text{p}}$ does not appear, and the summation is restricted to $j^{\prime }\neq i^{\prime }$ for the $J_{j^{\prime }}^{\text{p}}$ operator. The analogous summation in Eq. (6) is not restricted because the usual Hartree–Fock formulation includes the self-Coulomb and self-exchange terms, such that the Fock operator is the same for each orbital. Including the self-Coulomb and self-exchange terms does not change the Hartree–Fock energy because they cancel exactly. Thus, we can also add the self-Coulomb and self-exchange terms for each nuclear Fock operator in the nuclear Hartree product representation [Eq. (7)] without changing the resulting energy. We will perform calculations using the nuclear Hartree product representation both without and with these nuclear self-interaction terms, denoted as HP and HP*, respectively, to analyze the convergence behavior.

### B. NEO density functional theory

An extension of the Hohenberg–Kohn theorems and Kohn–Sham formalism to multicomponent systems^26–28^ has been implemented within the NEO-DFT framework.^9,12,29,30^ In this approach, the NEO energy depends on both the electron and proton densities, $\rho^{\text{e}}$ and $\rho^{\text{p}}$, respectively. This formulation includes three types of functionals. $E_{\text{exc}}\left[\rho^{\text{e}}\right]$ is the electron–electron exchange–correlation functional and can be chosen to be any of the previously developed functionals from conventional electronic DFT. $E_{\text{epc}}\left[\rho^{\text{e}},\rho^{\text{p}}\right]$ is the electron–proton correlation functional and is essential for capturing even qualitatively accurate proton densities and zero-point energies. The most commonly used electron–proton correlation functional is the epc17-2 functional.^10,11^ $E_{\text{pxc}}\left[\rho^{\text{p}}\right]$ is the proton–proton exchange–correlation functional. As mentioned above, proton–proton exchange and correlation energies are negligible for molecular systems.^2^ In practice, $E_{\text{pxc}}\left[\rho^{\text{p}}\right]$ is usually chosen to be the exact proton–proton Hartree–Fock exchange to eliminate self-interaction error. Thus, the discussion of the nuclear Slater determinant and Hartree product representations in Sec. II A also pertains to NEO-DFT. In the NEO-DFT framework, the nuclear Fock operator in Eqs. (6) or (7) will also include a term related to the electron–proton correlation functional, $E_{\text{epc}}\left[\rho^{\text{e}},\rho^{\text{p}}\right]$.

### C. Simultaneous optimization

Equations (3) and (4) can be solved iteratively either in an alternating, stepwise fashion or simultaneously. The simultaneous optimization algorithm was first introduced using the DIIS solver^14,15^ but is generalizable to other types of optimization schemes. In the simultaneous DIIS solver, we define an error matrix whose elements are the sums of inner products of electronic and nuclear error vectors, $\left\langle e_{k}^{\text{e}}|e_{l}^{\text{e}}\right\rangle +\left\langle e_{k}^{\text{p}}|e_{l}^{\text{p}}\right\rangle$, where k,l∈{1,2,…,m} and m is the DIIS subspace size. Here, $e_{k}^{\text{e}}\left(e_{k}^{\text{p}}\right)$ is the electronic (nuclear) error vector constructed as the commutator of the electronic (nuclear) Fock and electronic (nuclear) density matrices at the kth iteration. The error matrix is used to solve a least-squares minimization problem to yield a set of coefficients c for constructing the extrapolated Fock matrices as linear combinations of the Fock matrices from previous iterations,

(8) $$F_{\text{new}}^{\text{e}}=\sum_{k=1}^{m}c_{k}F_{k}^{\text{e}},$$

(9) $$F_{\text{new}}^{\text{p}}=\sum_{k=1}^{m}c_{k}F_{k}^{\text{p}}.$$

These new Fock matrices are diagonalized to obtain a new set of electronic and nuclear molecular orbital coefficients, and this whole procedure is repeated until the norms for both electronic and nuclear error vectors fall below a certain convergence threshold.

Convergence difficulties may sometimes arise for pathological cases and may require the use of various techniques such as damping^31^ and level-shifting.^32^ Here, we broadly define difficult convergence to encompass cases where convergence leads to a stationary point that is not the global minimum and cases that fail to converge altogether. The DIIS solver is usually more prone to these convergence instabilities than second-order optimization SCF techniques, which were developed to remedy some of these issues but require evaluation of the orbital Hessian. The simplest second-order optimization technique is the Newton–Raphson approach,

(10) $$B_{k+1}s_{k+1}=-g_{k+1},$$

which uses both the Hessian B and gradient g to form an optimal step s. At each iteration, the step is used to optimize the electronic (nuclear) molecular orbital coefficients $C^{\text{e}(\text{p})}$ via a unitary transformation,

(11) $$C_{k+1}^{\text{e}(\text{p})}=C_{k}^{\text{e}(\text{p})}\text{exp}\left(\kappa^{\text{e}\left(\text{p}\right)}\right),$$

where κ is a skew-symmetric, orbital rotation matrix defined as $\kappa =\text{Skew}\left(s_{k+1}\right)$. The Newton–Raphson approach often leads to a quadratic convergence of the orbital rotation parameters, but it suffers from two main drawbacks that limit its practical application. First, it requires computation of the full molecular orbital Hessian for each iteration, which can be computationally prohibitive. Second, the molecular orbital Hessian is typically not positive definite at the start of the SCF procedure, which is problematic as it can result in a step uphill in energy.

These drawbacks can be remedied using quasi-Newton–Raphson solvers that employ the gradients from previous iterations to form an approximate Hessian B using a recursive scheme such as the Broyden–Fletcher–Goldfarb–Shanno (BFGS) algorithm.^33^ This method avoids explicit evaluation of the molecular orbital Hessian, and the BFGS Hessian is maintained to be positive definite. The GDM solver is one of the most robust quasi-Newton–Raphson variants for orbital optimization because it improves upon the BFGS Hessian by taking into account geometric considerations in orbital rotation space, enabling steps along geodesic directions as opposed to Euclidean directions, and employs an automated switching between line search and trust radius approaches to fine-tune the orbital rotation step. The extension of the simultaneous optimization algorithm to the GDM solver as well as to other quasi-Newton–Raphson solvers is straightforward. All these solvers require the gradient to be expressed as

(12) g=gegp,

where ${\left.g_{pq}^{\text{e}}\equiv \frac{\partial E}{\partial \kappa_{pq}^{\text{e}}}\right|}_{\kappa^{\text{e}}=0}$ and ${\left.g_{PQ}^{\text{p}}\equiv \frac{\partial E}{\partial \kappa_{PQ}^{\text{p}}}\right|}_{\kappa^{\text{p}}=0}$ are the first-order variations of the NEO energy with respect to electronic and nuclear orbital rotations, respectively.

Another approach for ensuring positive-definiteness is to diagonalize a scaled augmented Hessian matrix,

(13) 0αgTαgH1x~(α)=μ1x~(α),

where the augmented Hessian is constructed using both g and the exact nuclear–electronic molecular orbital Hessian H, which takes the form of the following block matrix:

(14) $$H=\left(\begin{array}{ll} H^{\text{ee}} & H^{\text{ep}}\\ H^{\text{pe}} & H^{\text{pp}} \end{array}\right).$$

This procedure results in a level-shifted Newton–Raphson equation similar in form to Eq. (10),

(15) $$\left(H-\mu I\right)\frac{1}{\alpha }\tilde{x}\left(\alpha \right)=-g,$$

where μ is a level-shift parameter, $\tilde{x}(\alpha )$ is the step or update vector, and I is the identity matrix. In practice, many augmented Hessian approaches avoid the construction of the full molecular orbital Hessian but require subroutines that can evaluate the action of the Hessian on a set of trial vectors. Solution of Eq. (13) and determination of the parameters are accomplished through an iterative Davidson procedure^34^ for finding the lowest root of the augmented Hessian matrix. For each Hessian-vector product formation in the Davidson procedure, we regularly transform the trial vectors into the molecular orbital basis and then call subroutines that evaluate the NEO coupled-perturbed SCF equations. To avoid issues with overstepping and understepping, α is used as a tuning parameter to ensure $\tilde{x}(\alpha )$ is contained within a trust region, defining the TRAH solver. Molecular orbitals are updated using Eq. (11) with the orbital rotation matrix $\kappa =\text{Skew}(\tilde{x})$.

For convenience, the various simultaneous SCF schemes described in this subsection have been based on the nuclear Slater determinant representation. The extension of the DIIS and GDM solvers to the nuclear Hartree product representation is straightforward, but the TRAH nuclear Hartree product solver is less straightforward because it requires computing separate off-diagonal coupling blocks for Eq. (14) due to the distinguishability of the protons. Additional technical details about these extensions to the nuclear Hartree product representation are provided in Sec. 6 of the supplementary material. In this work, we focus on DIIS, GDM, and TRAH solvers for the nuclear Slater determinant representation and only the DIIS and GDM solvers for the nuclear Hartree product representation.

### D. Initial guess

All NEO-SCF calculations require an initial guess for the electronic and nuclear molecular orbitals. For NEO calculations, all existing initial guess variants developed for conventional electronic structure methods can be used for the electronic initial guess. Common variants include the core or one-electron guess obtained by diagonalizing an electronic Hamiltonian matrix constructed from the kinetic energy and classical nuclear interaction terms, as well as the superposition of atomic densities (SAD) guess, which constructs a block density matrix by summing together the converged atomic densities at each atomic nucleus.^35,36^ The core guess is known to be a poor starting guess, and most quantum chemistry codes recommend the SAD guess or a closely related variant.

For the nuclear initial guess options, we can similarly employ a nuclear core or nuclear SAD variant, as first reported by Reiher and co-workers.^20^ For comparative purposes, in this work, we consider a poor starting guess to be synonymous with the core guess and a good starting guess to be synonymous with the SAD guess. Note that concerns have been raised about multiple nuclei potentially localizing onto the same basis function center when a full $N^{\text{p}}$-nuclei core Hamiltonian matrix is diagonalized.^18^ To avoid these potential issues with overlapping nuclear centers and to ensure that we are using the same nuclear core guess for both nuclear Slater determinant and nuclear Hartree product calculations, we construct a block-diagonalized nuclear core guess suggested by Mata and co-workers^18^ for the nuclear Slater determinant representation. This problem cannot occur for the nuclear Hartree product representation because each quantum proton is expanded in its own protonic basis set. We also explored other initial guess types, as discussed further in the supplementary material.

## III. COMPUTATIONAL DETAILS

The methods discussed in Sec. II were implemented in a developer version of Q-Chem 6.3,^37^ which we used to perform all subsequent calculations. We elected to use the default settings for the DIIS and GDM algorithms, corresponding to no level-shifting or damping and the DIIS subspace size set to 15. For the TRAH calculations, we interfaced Q-Chem with the OpenTrustRegion library^24^ and used the default settings. After each orbital rotation step, we form a new approximate diagonal to the inverse orbital Hessian for the Davidson preconditioner. For all calculations in this work, we consider energies to be converged when the energy change between consecutive iterations is below 1.0 × 10^−8^ Hartree and when the DIIS error, or root-mean square (RMS) gradient in the case of the GDM and TRAH solvers, is below 10^−8^. All NEO-SCF calculations in the main text were performed using simultaneous optimization.

For the calculations presented in Sec. IV A, we assembled a test set of 92 molecules comprised of 17 water clusters along with a subset of molecules obtained from the W4-17 dataset.^38^ Only molecules with more than one hydrogen and two or more classical nuclei were retained from the W4-17 dataset, resulting in 75 molecules. The geometries for all molecules were optimized at the conventional Møller–Plesset second-order perturbation theory (MP2) level using the def2-TZVP electronic basis set^39^ until the maximum gradient component was below 3.0 × 10^−4^ Hartree/Bohr. These optimized geometries, which are provided in the supplementary material, were used for all subsequent NEO-HF convergence tests. All protons were treated quantum mechanically with NEO-HF using the def2-TZVP electronic basis set and the PB4-D protonic basis set.^40^ The NEO-DFT results for these geometries are provided in the supplementary material. In Sec. IV B, we present the number of iterations and calculation times for five protonated water clusters that were also part of the test set in Sec. IV A.

In Sec. IV C, we assess two challenging systems with NEO-DFT. The first system is an open-shell radical subunit of *E. coli* ribonucleotide reductase.^41–43^ The geometry used for the single-point energy calculations on this enzyme is a representative conformation obtained from a conventional DFT quantum mechanical/molecular mechanical (QM/MM) free energy string simulation of a proton-coupled electron transfer reaction between tyrosines Y730 and Y731, mediated by a nearby glutamate.^44^ We treat only the transferring proton quantum mechanically in our NEO-DFT single-point energy calculations, which also include the protein and solvent environment using electrostatic embedding.^45,46^ These calculations were initiated with SAD electronic and nuclear initial guesses, and the nuclear self-Coulomb and self-exchange terms were not included. These calculations were performed with the 6–31+G** electronic basis set^47–49^ and the PB4-F2 protonic basis set.^40^ The ωB97X-D electronic exchange–correlation functional^50^ was used, and either the epc17-2 electron–proton correlation functional^10,11^ or no electron–proton correlation (noepc) was used.

The second system studied in Sec. IV C is a closed-shell singlet uranium complex, UO_2_(OH)_4_. For this system, we first optimized the geometry using conventional DFT with the B3LYP exchange–correlation functional,^51,52^ in conjunction with the LANL2DZ electronic basis set and the fit-LANL2DZ pseudopotential.^53–55^ Our subsequent NEO-DFT calculations on this complex utilized the same electronic basis set, pseudopotential,^56^ and electronic exchange–correlation functional along with the epc17-2 electron–proton correlation functional, the PB4-D protonic basis set,^40^ and the SAD electronic and nuclear initial guesses. We treated all four protons quantum mechanically and used both the nuclear Slater determinant and nuclear Hartree product representations.

## IV. RESULTS AND DISCUSSION

### A. Nuclear Slater determinant and Hartree product comparisons for 92 molecules

**FIG. 1.**
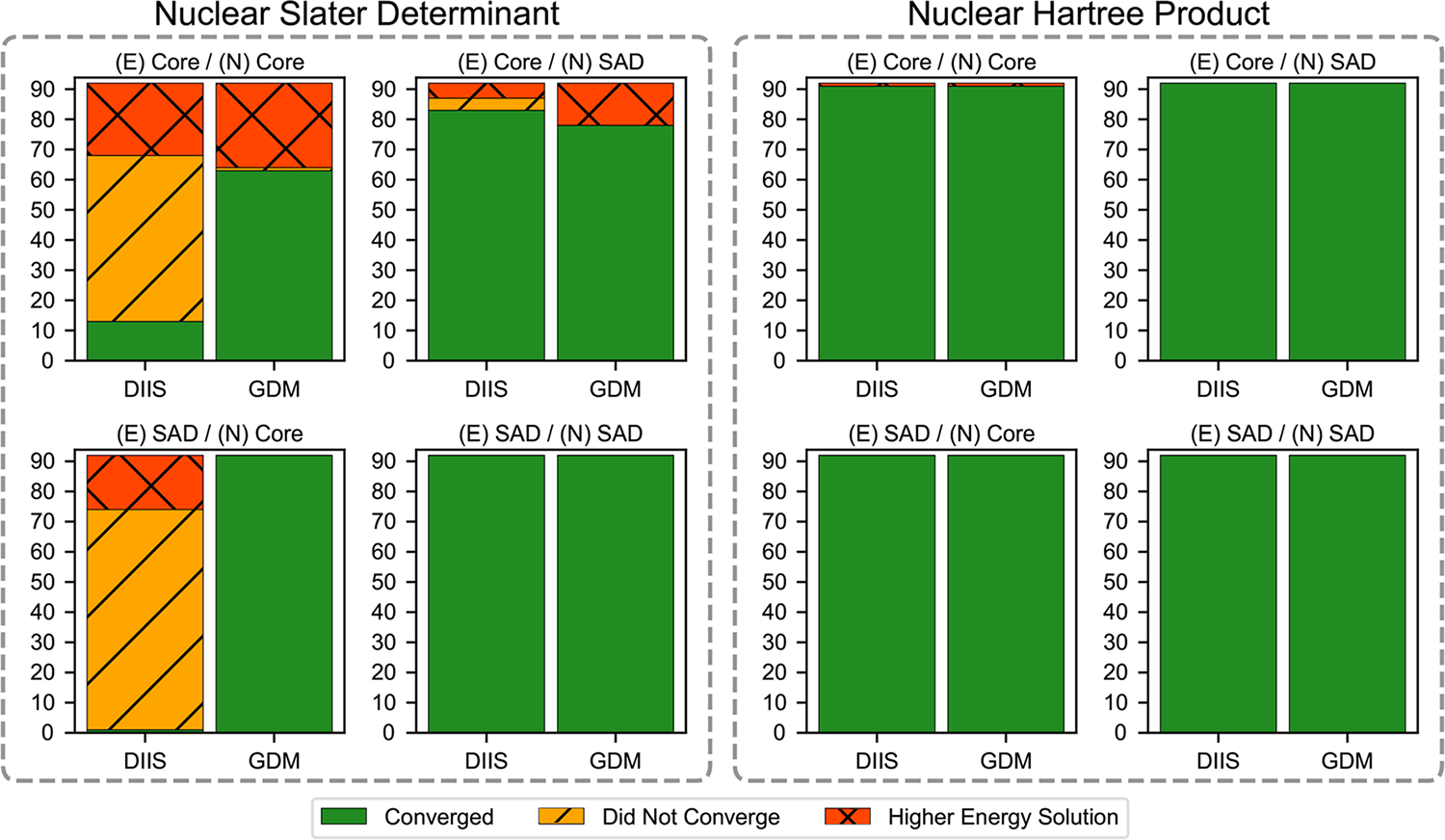
NEO-HF SCF convergence statistics for a set of 92 molecules with all protons quantized. Calculations were initiated using the core or SAD initial guess for the electronic (E) and nuclear (N) orbitals in conjunction with either the DIIS or GDM SCF solver for the nuclear Slater determinant or nuclear Hartree product representation. Each bar graph illustrates the number of cases where SCF converged to a higher-energy solution (red), did not converge (gold), or successfully converged to the purported lowest-energy minimum (green).

For our set of 92 molecules, we performed NEO-HF calculations using different initial guesses in conjunction with both DIIS and GDM solvers (Fig. 1). These calculations were carried out for both nuclear Slater determinant and nuclear Hartree product representations. Additional NEO-HF convergence statistics are provided in Tables S2–S8. Focusing first on the nuclear Slater determinant results, Fig. 1 shows that using a poor initial guess for the electronic subsystem, the nuclear subsystem, or both subsystems can result in numerous convergence instabilities. When a core guess is used, the DIIS solver leads to a higher-energy solution or fails to converge for some fraction of the molecules. When using a more robust solver, such as GDM, convergence is usually achieved, but the solution can be trapped in a higher-energy solution, which could be a local minimum or a saddle point, when a core guess is used for the electronic orbitals. This behavior may be remedied in part by using a hybrid DIIS–GDM solver, but such a hybrid scheme is not effective across all cases (Table S2).

These convergence issues are not present when good initial guesses are used. Moreover, these convergence issues are absent in all of the conventional electronic HF calculations except for one case (Table S1), indicating that the NEO method is more sensitive to the initial guess. These findings also hold for NEO-DFT calculations that include electron–proton correlation as well as electronic exchange and correlation (Fig. S2). Interestingly, this heightened sensitivity to the choice of initial guess is mitigated when the nuclear Hartree product representation is used. Even with poor starting guesses, we observed no convergence instabilities for the DIIS or GDM solver when the nuclear Hartree product representation was used, except for one case.

**FIG. 2.**
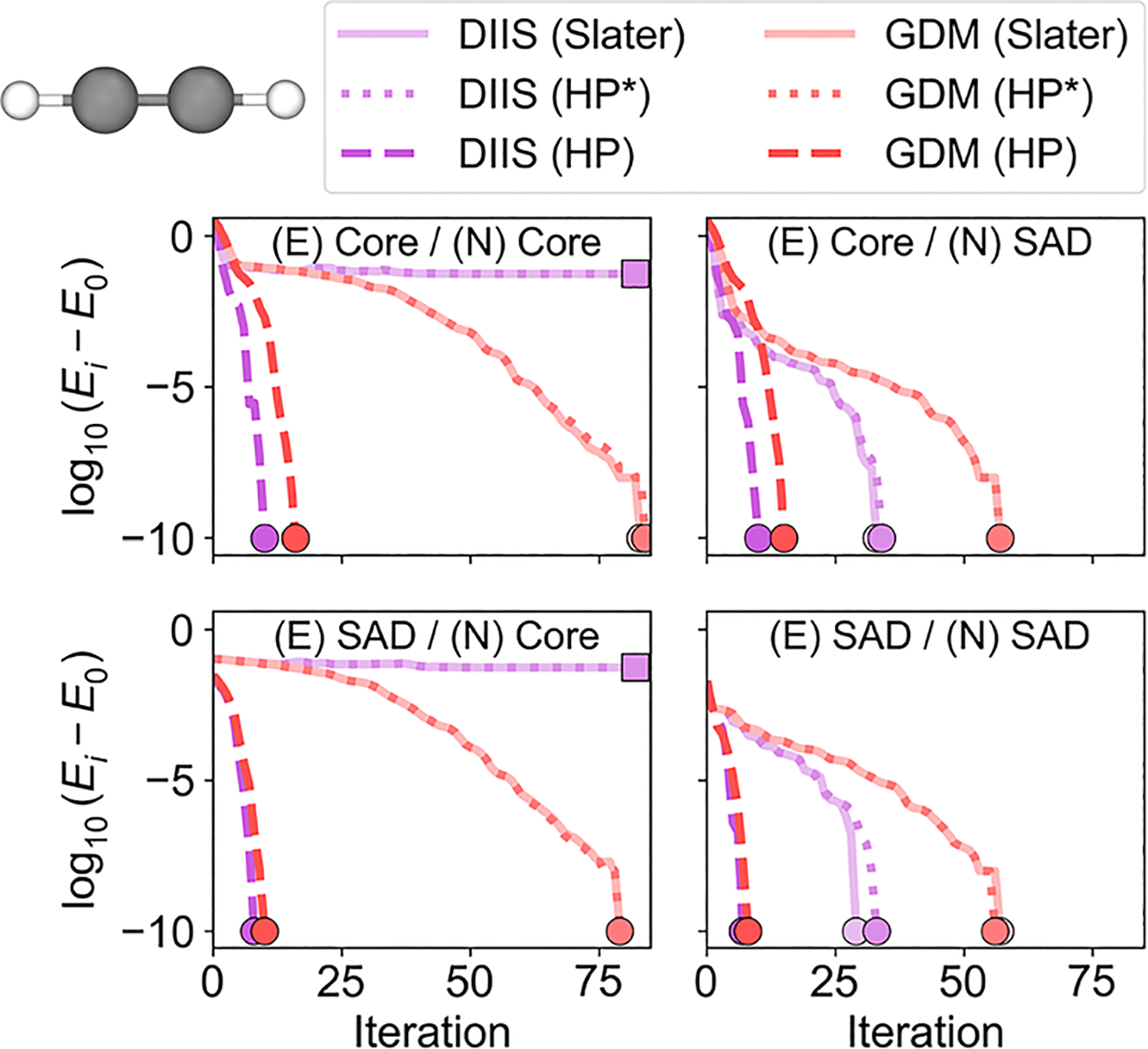
NEO-HF energy convergence behavior for an acetylene molecule, where its two protons are treated quantum mechanically using the nuclear Hartree product representation or the nuclear Slater determinant representation. Here, $E_{i}$ and $E_{0}$ are the energy at iteration i and the purported lowest-energy solution, respectively. Calculations were performed using simultaneous DIIS and GDM solvers with different initial guess combinations. When a nuclear core guess was used, the DIIS solver with the nuclear Slater determinant representation (solid lines) failed to converge to the correct solution (indicated by square markers). When the nuclear Hartree product representation with the inclusion of self-Coulomb and self-exchange terms (dotted lines and denoted as HP*) was used, the convergence trends were nearly identical to those obtained with the nuclear Slater determinant representation (solid lines). When the nuclear Hartree product representation (dashed lines and denoted as HP) was used, all calculations converged to the same energy (indicated by circle markers), independent of initial guess and SCF solver.

As a further investigation, we analyzed the convergence behavior of a single molecule from our dataset (Fig. 2). For the nuclear Slater determinant representation, the DIIS solver converged to a higher-energy solution when a nuclear core guess was used, whereas the GDM solver was able to find a lower-energy solution. When a nuclear SAD guess was used, both the DIIS and GDM solvers converged, but the GDM solver required approximately double the number of SCF iterations. When the nuclear Hartree product representation was employed, both the DIIS and GDM solvers converged in a comparable number of iterations, independent of the choice of initial guesses (dashed lines in Fig. 2). Moreover, these calculations converged in far fewer iterations than did calculations using the nuclear Slater determinant representation. The average number of iterations for calculations on the set of 92 molecules using these various approaches is given in Table S4.

In order to pinpoint the source of additional complexity, we added the nuclear self-Coulomb and self-exchange terms for every proton in the nuclear Hartree product representation. Adding these terms does not impact the total energy due to exact cancellation. Interestingly, these results show that the convergence behavior is nearly identical to that observed for the nuclear Slater determinant representation (compare the solid and dotted lines, which are nearly superimposed, in Fig. 2). This test suggests that these nuclear self-interaction terms are a significant contributor to the overall optimization complexity as well as the sensitivity to the initial guess in NEO calculations. To further test this idea, we performed calculations on the full set of molecules in Fig. 1 using the nuclear Hartree product representation with the inclusion of nuclear self-Coulomb and self-exchange terms (Table S7), as well as calculations treating only a single proton quantum mechanically with or without these nuclear self-interaction terms (Fig. S1). Many of these test calculations show convergence instabilities when the nuclear self-interaction terms are included. The calculations for systems with only a single quantum proton indicate that these issues are not limited to systems with multiple quantum protons.

### B. Computational cost comparisons for protonated water clusters

**FIG. 3.**
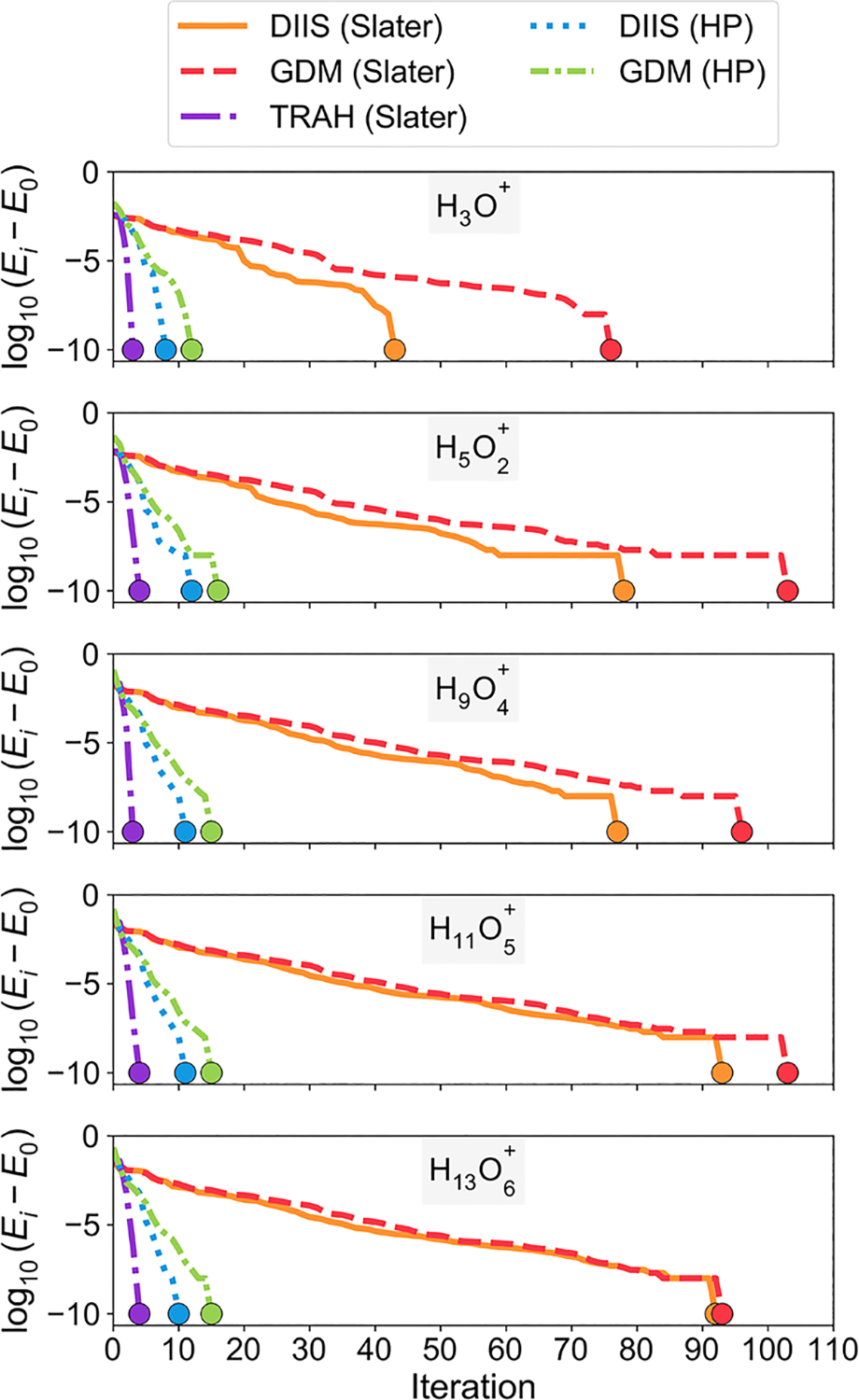
NEO-HF energy convergence behavior for a set of protonated water clusters of increasing size with all protons treated quantum mechanically. SAD electronic and nuclear guesses were used for all calculations. The various line types show the results for the nuclear Slater determinant representation with the DIIS, GDM, and TRAH solvers and the results for the nuclear Hartree product representation with the DIIS and GDM solvers. For each system, all optimization algorithms converged to the same energy (indicated by circle markers). Wall times for these calculations performed on the same CPU are provided in Table I.

We further illustrate the simplification of the optimization problem when the nuclear Hartree product representation is used, thereby eliminating the nuclear self-interaction terms, by investigating the number of SCF iterations and overall calculation times for protonated water clusters. In Fig. 3, we show the convergence behavior for protonated water clusters using various SCF solver algorithms and employing either a nuclear Slater determinant representation or a nuclear Hartree product representation. For the nuclear Slater determinant representation, the DIIS solver converged in a smaller number of iterations than did the GDM solver for all five water molecules, although the difference is minimal in some cases. The TRAH solver converged in the smallest number of iterations compared to all other solvers shown in Fig. 3, but each iteration is more costly.

In comparison to the nuclear Slater determinant results, the number of iterations is significantly reduced for both the DIIS and GDM solvers when the nuclear Hartree product representation is employed. In NEO-HF, the one-particle Fock operator defined in Eq. (7) for each proton in the nuclear Hartree product representation is a linear operator that depends on only the mean field of the electrons and other quantum protons. In other words, the nuclear Fock matrix for each proton [Eq. (4)] can be diagonalized in a single step and does not require an iterative procedure. This property of the nuclear Hartree product representation is likely the reason behind the substantial reduction in the number of iterations compared to those required for the nuclear Slater determinant representation. Further evidence is provided by the single quantum proton calculations shown in Table S9 and Fig. S1. These calculations show that omission of the nuclear self-Coulomb and self-exchange terms also significantly decreases the number of iterations required for convergence for systems with only a single quantum proton. Note that although Eq. (4) can be solved in a single iteration, in practice, it is solved more than once because it is coupled to Eq. (3), which must be solved iteratively.

Since a systematic cost-per-iteration evaluation of the various approaches is not straightforward, we provide the overall NEO-SCF calculation times for the data shown in Fig. 3. For the nuclear Slater determinant representation results in Table I, the DIIS solver is the most efficient, and the TRAH solver is the least efficient. For the nuclear Hartree product representation, the DIIS and GDM solvers converged around five to ten times faster than did their Slater determinant counterparts. Although the quantitative timings for the three SCF algorithms could be influenced by the choice of algorithm hyperparameters, construction of a different Davidson initial guess and preconditioner, as well as other technical aspects, the key finding is the speed-up attributed to the absence of nuclear self-Coulomb and self-exchange terms in the nuclear Hartree product representation. The primary cost savings for the Hartree product representation are due to the reduction in the number of NEO-SCF iterations, as shown in Fig. 3. However, the cost-per-iteration evaluation is also slightly more efficient when the exchange terms do not need to be evaluated, as shown in Table S11.

### C. Difficult convergence cases

In this section, we illustrate that NEO-SCF convergence difficulties can also arise due to factors unrelated to nuclear self-Coulomb and self-exchange terms. We present two examples calculated with NEO-DFT to show the need for robust algorithms such as GDM and TRAH. We first perform calculations on the enzyme ribonucleotide reductase, treating one proton quantum mechanically without nuclear self-Coulomb and self-exchange terms. We then consider the UO_2_(OH)_4_ complex, treating all of its protons quantum mechanically within the nuclear Slater determinant and nuclear Hartree product representations.

**FIG. 4.**
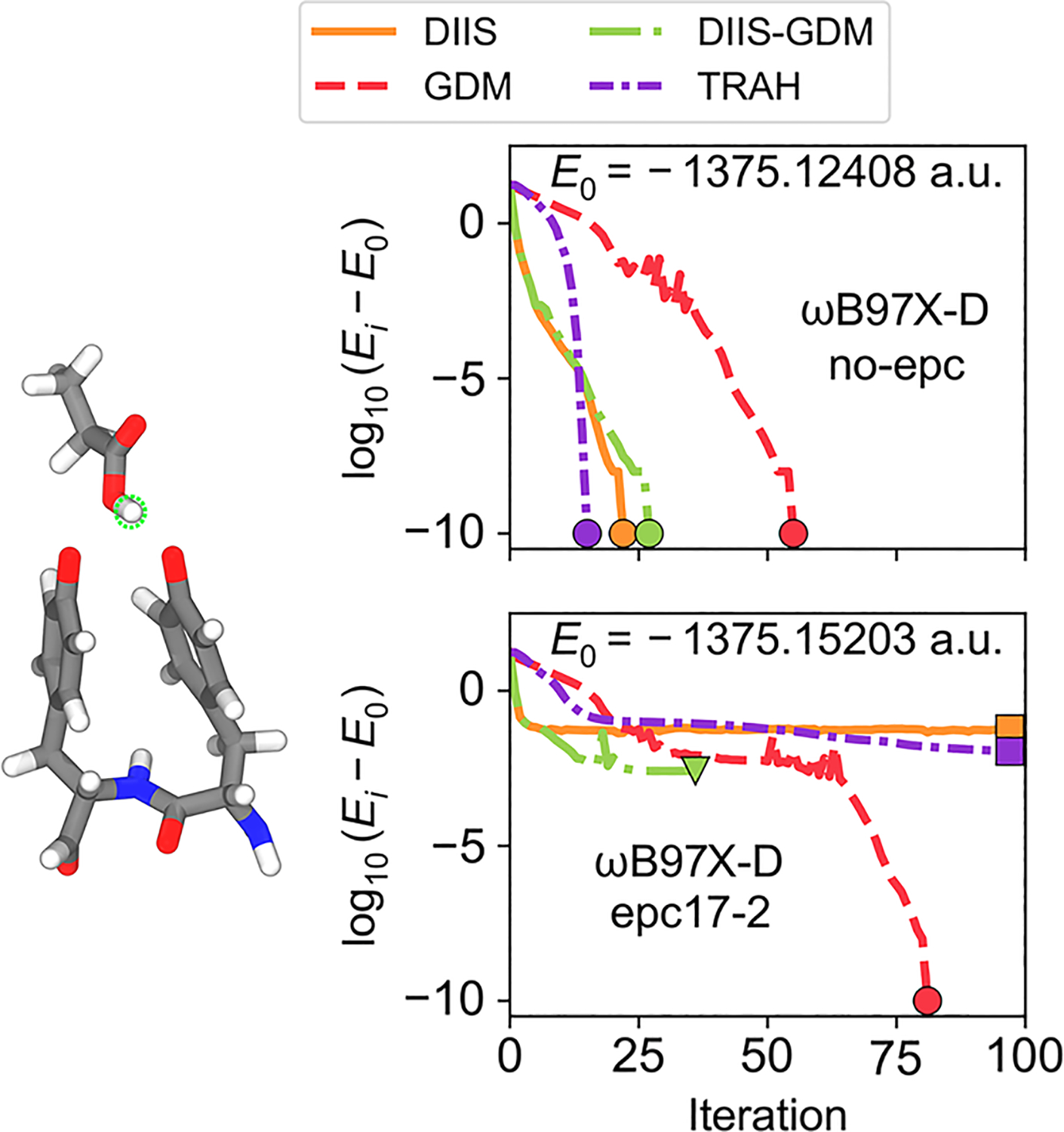
NEO-DFT energy convergence behavior for a representative conformation obtained from a QM/MM free energy string simulation for the proton-coupled electron transfer reaction between tyrosines Y730 and Y731 in the enzyme ribonucleotide reductase. The QM region is shown on the left, and the panels on the right show the NEO-DFT energy convergence behavior, where the transferring proton (circled in green on the conformation) is treated quantum mechanically, and the protein and solvent environment are included with electrostatic embedding. For NEO-DFT with no-epc (top panel), the calculations converged to the same lowest-energy solution (indicated by circle markers), $E_{0}$. For NEO-DFT with epc17-2 (bottom panel), the DIIS and TRAH solvers failed to converge (indicated by square markers), the hybrid DIIS–GDM solver converged to a higher-energy solution (indicated by a triangle marker), and the GDM solver converged to the purported lowest-energy solution (indicated by a circle marker), $E_{0}$, which is different than the value in the top panel due to the incorporation of electron–proton correlation.

Figure 4 shows the convergence behavior for NEO-DFT calculations on the enzyme ribonucleotide reductase with the epc17-2 electron–proton correlation functional^10,11^ and without this functional (no-epc). In this example, all NEO-SCF solvers converged for the no-epc case (top panel of Fig. 4), but only the GDM solver converged to the purported lowest-energy minimum when electron–proton correlation was included using the epc17-2 functional (bottom panel). A hybrid DIIS–GDM solver also converged but to a higher-energy stationary point. Interestingly, both the DIIS and TRAH solvers were unable to converge. However, it is possible that tuning the trust radius hyperparameters or changing the DIIS subspace size in combination with level-shifting could yield convergence.

**FIG. 5.**
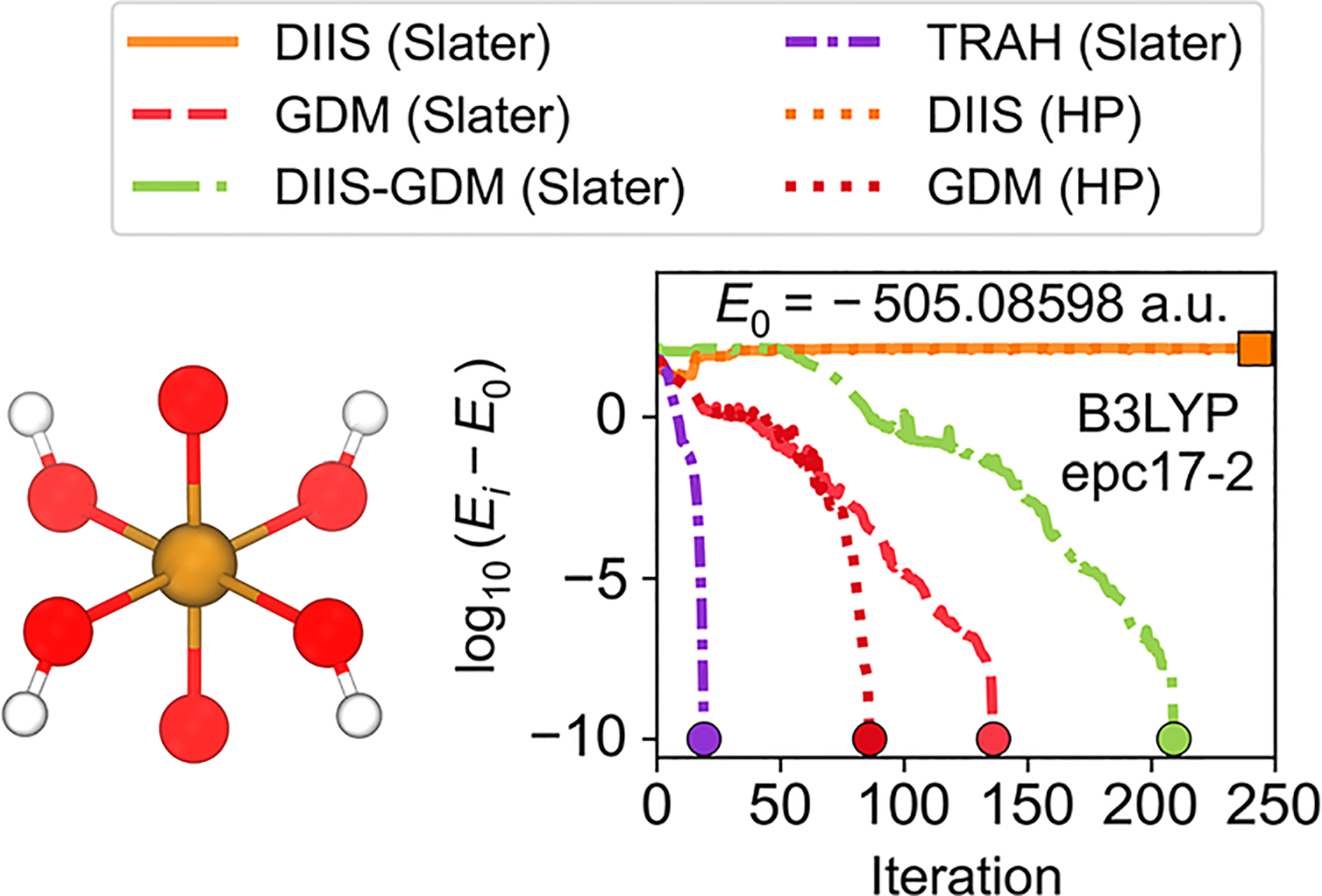
NEO-DFT/epc17-2 energy convergence behavior for the UO_2_(OH)_4_ molecule with all four protons treated quantum mechanically. The DIIS solver failed to converge (indicated by a square marker), whereas the GDM, hybrid DIIS–GDM, and TRAH solvers converged to the same solution, $E_{0}$ (indicated by circle markers). The nuclear Hartree product representation required fewer iterations than the nuclear Slater determinant representation using the GDM solver (compare the red dashed and darker red dotted lines).

Figure 5 shows the convergence behavior for the UO_2_(OH)_4_ complex. This complex is known to have a complicated electronic structure and demonstrates erratic convergence behavior for conventional electronic structure calculations.^23,24,57^ We performed these NEO-DFT calculations with the epc17-2 electron–proton correlation functional. We found that the GDM, hybrid DIIS–GDM, and TRAH solvers converged to the same solution, whereas the DIIS solver failed to converge. Similar behavior was observed for the nuclear Slater determinant and Hartree product representations using the DIIS and GDM solvers, although fewer iterations were required to achieve convergence with the GDM solver for the nuclear Hartree product representation. When multiple approaches lead to the same lowest-energy solution, as shown in Fig. 5, it is likely that this solution is a minimum, although a stability analysis of the orbital Hessian^58^ would be required for confirmation. The analogous conventional electronic DFT calculations show convergence problems for the DIIS solver (Fig. S3), indicating that convergence for this system is complicated due to the underlying complexity of the electronic structure. These applications to ribonucleotide reductase and UO_2_(OH)_4_ highlight the importance of access to a broad selection of NEO-SCF algorithms for handling challenging systems.

## V. CONCLUSION

In this work, we showed that a significant reduction in the complexity and computational cost of NEO-SCF optimizations can be achieved when the nuclear self-Coulomb and self-exchange terms are not included. This approach is valid for systems with only one quantum proton or for systems with multiple quantum protons described by the nuclear Hartree product representation. This discovery deepens our understanding of the primary reason for the significant computational cost savings provided by the nuclear Hartree product representation. In the original work,^21^ it was proposed that the reduction in the number of integrals and the simpler matrix diagonalizations were the main reasons for the cost reduction. Herein, we showed that the major cost reduction is due to the substantial decrease in the number of iterations required for convergence when the nuclear self-Coulomb and self-exchange terms are omitted. For systems with multiple quantum protons, treating the nuclear wave function as a Hartree product rather than a Slater determinant does not change the NEO ground-state energy because the proton–proton exchange energy is typically many orders of magnitude smaller than the SCF energy convergence threshold. Thus, this work leads to the recommendation of the nuclear Hartree product representation for NEO-SCF calculations. We also extended the simultaneous optimization algorithm from the DIIS solver to both the GDM and TRAH solvers and showed that they can be useful for converging difficult problems. These findings will serve as guidance for performing more efficient and robust NEO calculations.

## Supplementary Material

Supplementary Material

Geometries

The supplementary material encompasses additional conventional Hartree–Fock convergence statistics, additional NEO-HF convergence statistics for systems with multiple quantum protons, NEO-HF convergence statistics for systems with a single quantum proton, NEO-DFT convergence statistics for systems with multiple quantum protons, conventional DFT calculations on UO_2_(OH)_4_, and additional technical and timing information.

## Figures and Tables

**TABLE I.** NEO-SCF wall times (s) for the water cluster calculations shown in Fig. 3, calculated using an Intel(R) Xeon(R) Gold 6242R CPU with 32 cores. Results with the nuclear Slater determinant representation and the nuclear Hartree product representation are given.

	Slater determinant			Hartree product	
System	DIIS	GDM	TRAH	DIIS	GDM
H_3_O^+^	11.7	22.5	113.8	1.7	2.0
H_5_O_2_^+^	23.4	40.6	69.0	3.8	5.1
H_9_O_4_^+^	65.5	110.3	121.1	12.1	16.4
H_11_O_5_^+^	124.5	166.9	238.9	21.0	28.0
H_13_O_6_^+^	141.0	233.4	389.6	28.5	38.6

## Data Availability

The geometries used for all calculations are provided in the supplementary material. In addition, all Q-Chem output files used to support these findings will be openly available on Zenodo at https://doi.org/10.5281/zenodo.18227488.
