# Supplementary material for: Efficient and robust optimization of nuclear and electronic orbitals within the nuclear Hartree product representation
Source: J Chem Phys. Author manuscript; Available in PMC 2026 Jun 8. (PMC13246137; doi:10.1063/5.0308634)
Supplement: Supplementary Material [file NIHMS2179564-supplement-Supplementary_Material.pdf]

# Supplementary Material

# Efficient and robust optimization of nuclear and electronic orbitals within the nuclear Hartree product representation

Mathew Chow,[†,‡] Eno Paenurk,[‡] and Sharon Hammes-Schiffer*[,†,‡]

†*Department of Chemistry, Yale University, New Haven, CT 06520, United States*

‡*Department of Chemistry, Princeton University, Princeton, NJ 08544, United States*

E-mail: shs566@princeton.edu

# Contents

**1. Conventional Hartree–Fock convergence statistics** **S3**

**2. Additional multiple quantum proton NEO-HF convergence statistics** **S3**

**3. NEO-HF convergence statistics for single quantum proton** **S8**

**4. Multiple quantum proton NEO-DFT convergence statistics** **S10**

**5. Conventional density functional theory calculations on $UO_2(OH)_4$** **S12**

**6. Additional Technical Information** **S13**

**7. Additional Timing Information** **S15**

References **S16**

# 1. Conventional Hartree–Fock convergence statistics

For the set of 92 molecules studied in the main text, conventional electronic Hartree–Fock (HF) calculations on the same geometries and utilizing the same def2-TZVP electronic basis set and SCF convergence criteria were performed. The convergence statistics in Table S1 indicate that these calculations are not strongly influenced by the choice of initial guess or SCF solver.

Table S1: Conventional electronic HF SCF convergence statistics for a set of 92 molecules.

| | DIIS | | GDM | | DIIS-GDM | |
|---|---|---|---|---|---|---|
| Initial Guess | Higher Energy Solution | Did Not Converge | Higher Energy Solution | Did Not Converge | Higher Energy Solution | Did Not Converge |
| SAD | 0 | 0 | 0 | 0 | 0 | 0 |
| Core | 0 | 0 | 1 | 0 | 0 | 0 |

# 2. Additional multiple quantum proton NEO-HF convergence statistics

In Figure 1 in the main text, calculations were initiated with either an electronic (nuclear) core guess or electronic (nuclear) SAD guess. In this section, we show results for two additional NEO-SCF initial guesses: a converged result from a conventional electronic calculation for the electronic initial guess and a nuclear tight guess corresponding to the tightest protonic basis function for each quantum nucleus for the nuclear initial guess. This nuclear tight guess was introduced in the ChronusQ software package,[1] and a closely related variant was developed by Lehtola and coworkers in the OpenOrbitalOptimizer library.[2] The NEO-HF results obtained with the simultaneous optimization approach using the nuclear Slater determinant representation or the nuclear Hartree product representation are given in Tables S2 and S3, respectively. These tables were used to generate Figure 1 in the main text. The corresponding average number of simultaneous NEO-HF iterations for the electronic SAD guess and

nuclear SAD guess calculations are given in Table S4. The NEO-HF results obtained with the stepwise optimization approach are given in Tables S5 and S6, and the NEO-HF results obtained with the simultaneous optimization approach using the nuclear Hartree product representation calculations with inclusion of nuclear self-Coulomb and self-exchange terms are given in Tables S7 and S8.

Table S2: NEO-HF simultaneous calculations using the nuclear Slater determinant representation for a set of 92 molecules with all protons quantized.

| | | DIIS | | GDM | | DIIS-GDM | |
|---|---|---|---|---|---|---|---|
| Electronic Initial Guess | Nuclear Initial Guess | Higher Energy Solution | Did Not Converge | Higher Energy Solution | Did Not Converge | Higher Energy Solution | Did Not Converge |
| SCF | SAD | 0 | 0 | 0 | 0 | 0 | 0 |
| | Tight | 2 | 0 | 0 | 0 | 0 | 0 |
| | Core | 23 | 67 | 0 | 0 | 0 | 0 |
| SAD | SAD | 0 | 0 | 0 | 0 | 0 | 0 |
| | Tight | 0 | 0 | 0 | 0 | 0 | 0 |
| | Core | 18 | 73 | 0 | 0 | 0 | 0 |
| Core | SAD | 5 | 4 | 14 | 0 | 6 | 0 |
| | Tight | 8 | 0 | 26 | 1 | 6 | 2 |
| | Core | 24 | 55 | 28 | 1 | 8 | 0 |

Table S3: NEO-HF simultaneous calculations using the nuclear Hartree product representation for a set of 92 molecules with all protons quantized.

| | | DIIS | | GDM | |
|---|---|---|---|---|---|
| Electronic Initial Guess | Nuclear Initial Guess | Higher Energy Solution | Did Not Converge | Higher Energy Solution | Did Not Converge |
| SCF | SAD | 0 | 0 | 0 | 0 |
| | Tight | 0 | 0 | 0 | 0 |
| | Core | 0 | 0 | 0 | 0 |
| SAD | SAD | 0 | 0 | 0 | 0 |
| | Tight | 0 | 0 | 0 | 0 |
| | Core | 0 | 0 | 0 | 0 |
| Core | SAD | 0 | 0 | 0 | 0 |
| | Tight | 0 | 0 | 0 | 0 |
| | Core | 1 | 0 | 1 | 0 |

Table S4: Average number of simultaneous NEO-HF SCF iterations for a set of 92 molecules with all protons quantized, corresponding to the results shown in Figure 1 of the main text using data from Tables S2 and S3. Results obtained using the nuclear Slater determinant representation or the nuclear Hartree product representation for both the DIIS and GDM solvers with electronic and nuclear SAD initial guesses are given.

| Slater determinant | | Hartree product | |
|---|---|---|---|
| DIIS | GDM | DIIS | GDM |
| 90 | 154 | 20 | 24 |

Table S5: NEO-HF stepwise calculations using the nuclear Slater determinant representation for a set of 92 molecules with all protons quantized.

| | | DIIS | | GDM | |
|---|---|---|---|---|---|
| Electronic Initial Guess | Nuclear Initial Guess | Higher Energy Solution | Did Not Converge | Higher Energy Solution | Did Not Converge |
| SCF | SAD | 0 | 0 | 0 | 0 |
| | Tight | 41 | 8 | 0 | 0 |
| | Core | 84 | 4 | 0 | 0 |
| SAD | SAD | 0 | 0 | 0 | 0 |
| | Tight | 0 | 0 | 0 | 0 |
| | Core | 74 | 1 | 0 | 0 |
| Core | SAD | 23 | 0 | 1 | 0 |
| | Tight | 20 | 1 | 1 | 0 |
| | Core | 30 | 0 | 2 | 0 |

Table S6: NEO-HF stepwise calculations using the nuclear Hartree product representation for a set of 92 molecules with all protons quantized.

| | | DIIS | | GDM | |
|---|---|---|---|---|---|
| Electronic Initial Guess | Nuclear Initial Guess | Higher Energy Solution | Did Not Converge | Higher Energy Solution | Did Not Converge |
| SCF | SAD | 0 | 0 | 0 | 0 |
| | Tight | 0 | 0 | 0 | 0 |
| | Core | 0 | 0 | 0 | 0 |
| SAD | SAD | 0 | 0 | 0 | 0 |
| | Tight | 0 | 0 | 0 | 0 |
| | Core | 0 | 0 | 0 | 0 |
| Core | SAD | 0 | 0 | 0 | 0 |
| | Tight | 0 | 0 | 0 | 0 |
| | Core | 0 | 0 | 0 | 0 |

Table S7: NEO-HF simultaneous calculations using the nuclear Hartree product representation with inclusion of nuclear self-Coulomb and self-exchange terms for a set of 92 molecules with all protons quantized.

| | | DIIS | | GDM | |
|---|---|---|---|---|---|
| Electronic Initial Guess | Nuclear Initial Guess | Higher Energy Solution | Did Not Converge | Higher Energy Solution | Did Not Converge |
| SCF | SAD | 0 | 0 | 0 | 0 |
| | Tight | 3 | 0 | 0 | 0 |
| | Core | 18 | 72 | 0 | 0 |
| SAD | SAD | 0 | 0 | 0 | 0 |
| | Tight | 0 | 0 | 0 | 0 |
| | Core | 18 | 73 | 0 | 0 |
| Core | SAD | 0 | 0 | 0 | 0 |
| | Tight | 0 | 0 | 0 | 0 |
| | Core | 16 | 70 | 0 | 0 |

Table S8: NEO-HF stepwise calculations using the nuclear Hartree product representation with inclusion of nuclear self-Coulomb and self-exchange terms for a set of 92 molecules with all protons quantized.

| | | DIIS | | GDM | |
|---|---|---|---|---|---|
| Electronic Initial Guess | Nuclear Initial Guess | Higher Energy Solution | Did Not Converge | Higher Energy Solution | Did Not Converge |
| SCF | SAD | 0 | 0 | 0 | 0 |
| | Tight | 90 | 0 | 0 | 0 |
| | Core | 91 | 1 | 0 | 0 |
| SAD | SAD | 0 | 0 | 0 | 0 |
| | Tight | 6 | 0 | 0 | 0 |
| | Core | 92 | 0 | 0 | 0 |
| Core | SAD | 48 | 0 | 0 | 0 |
| | Tight | 46 | 0 | 0 | 0 |
| | Core | 52 | 0 | 0 | 0 |

# 3. NEO-HF convergence statistics for single quantum proton

The analogous NEO-HF calculations as those presented in Figure 1 in the main text were performed but treating only one proton quantum mechanically for each molecule. These calculations indicate convergence instabilities when nuclear self-Coulomb and self-exchange terms are included (Figure S1). The average numbers of NEO-HF iterations are given in Table S9.

Table S9: Average number of simultaneous NEO-HF SCF iterations for a set of 92 molecules with only a single proton quantized, corresponding to the results shown in Figure S1. Results obtained using both the DIIS and GDM solvers with electronic and nuclear SAD initial guesses, either including (with self-JK) or excluding (without self-JK) the self-Coulomb and self-exchange terms, are given.

| With self-JK | | Without self-JK | |
|---|---|---|---|
| DIIS | GDM | DIIS | GDM |
| 88 | 134 | 18 | 20 |

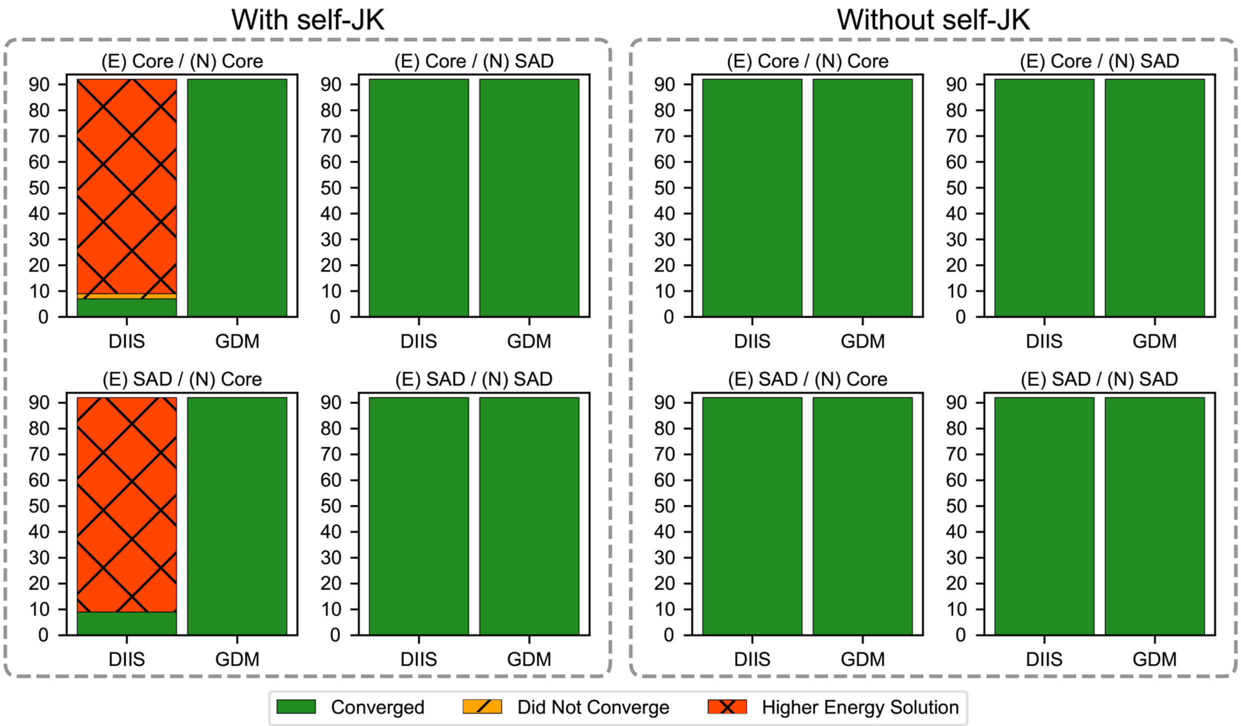


Figure S1: NEO-HF SCF convergence statistics for a set of 92 molecules with only one proton quantized. Calculations were initiated using the core or SAD initial guess for the electronic (E) and nuclear (N) orbitals in conjunction with either the DIIS or GDM SCF solver for the nuclear Slater determinant or nuclear Hartree product representation. Each bar graph illustrates the number of cases where SCF converged to a higher-energy solution (red), did not converge (gold), or successfully converged to the purported lowest-energy minimum (green).

## 4. Multiple quantum proton NEO-DFT convergence statistics

Analogous calculations on the set of 92 molecules using the same geometries as used for the NEO-HF calculations presented in Figure 1 in the main text were performed with NEO density functional theory (NEO-DFT). These calculations utilized the B3LYP electronic exchange-correlation functional, the epc17-2 electron–proton correlation functional, and the same basis sets as in the main text. These calculations indicate convergence instabilities in some cases, particularly for the core initial guess in conjunction with the nuclear Slater determinant representation (Figure S2). The average numbers of NEO-DFT iterations are given in Table S10.

Table S10: Average number of simultaneous NEO-DFT/epc17-2 SCF iterations for a set of 92 molecules with all protons quantized, corresponding to the results shown in Figure S2. Results obtained using the nuclear Slater determinant representation or the nuclear Hartree product representation for both the DIIS and GDM solvers with electronic and nuclear SAD initial guesses are given.

| Slater determinant | | Hartree product | |
|---|---|---|---|
| DIIS | GDM | DIIS | GDM |
| 74 | 114 | 51 | 31 |

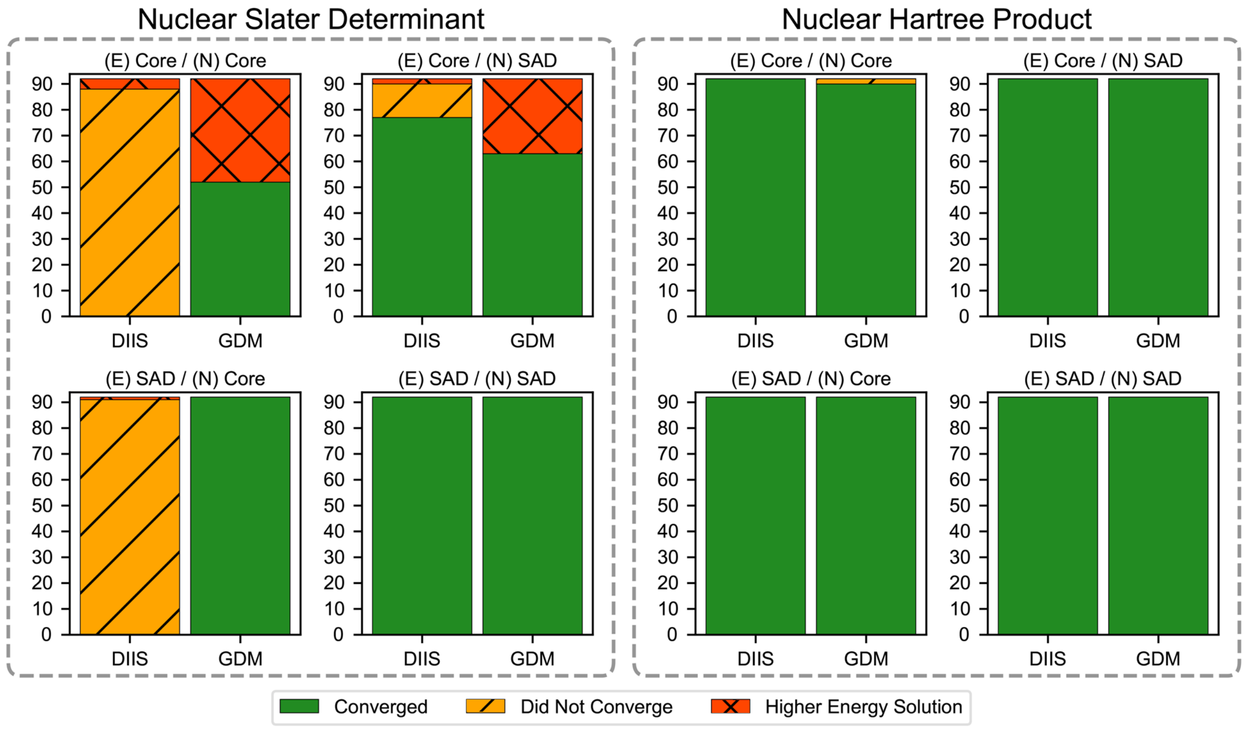


Figure S2: NEO-DFT SCF convergence statistics for a set of 92 molecules with all protons quantized. Calculations were initiated using the core or SAD initial guess for the electronic (E) and nuclear (N) orbitals in conjunction with either the DIIS or GDM SCF solver for the nuclear Slater determinant or nuclear Hartree product representation. Each bar graph illustrates the number of cases where SCF converged to a higher-energy solution (red), did not converge (gold), or successfully converged to the purported lowest-energy minimum (green).

# 5. Conventional density functional theory calculations on $UO_2(OH)_4$

The convergence behavior for conventional electronic DFT calculations on the same $UO_2(OH)_4$ geometry as used in the main text and utilizing the same electronic exchange-correlation functional (B3LYP) and electronic basis set (LANL2DZ/fit-LANL2DZ) is shown in Figure S3.

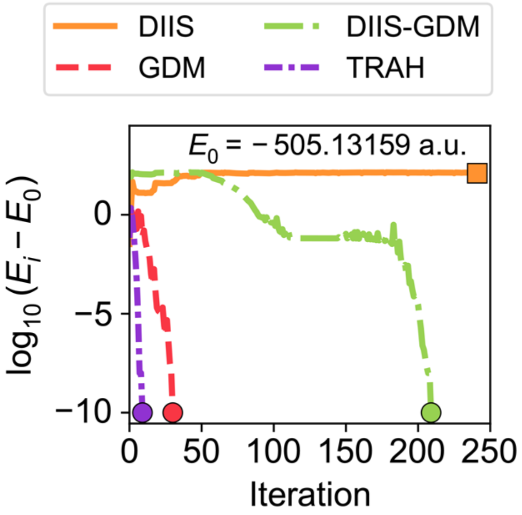


Figure S3: Conventional electronic DFT convergence behavior for the $UO_2(OH)_4$ molecule, analogous to the data shown in Figure 5 in the main text. The DIIS solver failed to converge (indicated by a square marker), whereas the GDM, hybrid DIIS-GDM, and TRAH solvers converged to the same solution, $E_0$ (indicated by circle markers).

## 6. Additional Technical Information

Equations 12 and 14 in the main text show the form of the orbital gradient and orbital Hessian in terms of the Slater determinant representation of nuclei. Here we show the generalization to $N$ distinguishable quantum nuclei. The analogous form of Eq. 12 for the orbital gradient considering indistinguishable electrons, denoted with a superscript e, and $N$ distinguishable nuclei, denoted with superscripts 1, 2, ..., $N$, is given by

$$\mathbf{g} = \begin{pmatrix} \mathbf{g}^{\mathrm{e}} \\ \mathbf{g}^{1} \\ \mathbf{g}^{2} \\ \vdots \\ \mathbf{g}^{N} \end{pmatrix} \tag{S1}$$

Similarly, the analagous form of Eq. 14 for the orbital Hessian is given by

$$\mathbf{H} = \begin{pmatrix} \mathbf{H}^{\mathrm{ee}} & \mathbf{H}^{\mathrm{e}1} & \mathbf{H}^{\mathrm{e}2} & \cdots & \mathbf{H}^{\mathrm{e}N} \\ \mathbf{H}^{1\mathrm{e}} & \mathbf{H}^{11} & \mathbf{H}^{12} & \cdots & \mathbf{H}^{1N} \\ \mathbf{H}^{2\mathrm{e}} & \mathbf{H}^{21} & \mathbf{H}^{22} & \cdots & \mathbf{H}^{2N} \\ \vdots & \vdots & \vdots & \ddots & \vdots \\ \mathbf{H}^{N\mathrm{e}} & \mathbf{H}^{N1} & \mathbf{H}^{N2} & \cdots & \mathbf{H}^{NN} \end{pmatrix} \tag{S2}$$

The simultaneous nuclear Hartree variant of the GDM solver requires computation of Eq. S1, which is straightforward to assemble. The simultaneous TRAH Hartree product solver requires both Eq. S1 and Eq. S2. Although Eq. S2 can in principle be straightforward to compute, it requires careful restructuring of the code in order to compute the off-diagonal coupling blocks of the Hessian for each distinguishable nucleus. Because of technical considerations, it was more straightforward to implement the simultaneous TRAH

Slater determinant solver at this time, but the Hartree product variant can be implemented in the future.

# 7. Additional Timing Information

Table S11: Wall times in seconds for water cluster calculations analogous to Figure 3 and Table 1 in the main text. All calculations in this table were performed on the same CPU, but it is a different type of CPU than that used for the calculations in Table 1 because these were performed at a later stage. Timings herein were obtained using the simultaneous DIIS solver for the nuclear Slater determinant and nuclear Hartree product representations. This table shows the total cost per iteration and the per iteration cost to compute the total nuclear Coulomb and exchange parts of the nuclear Fock matrix for the nuclear Slater determinant representation and the per iteration cost to compute the total nuclear Coulomb part of the nuclear Fock matrix for the nuclear Hartree product representation. Although the primary cost savings for the nuclear Hartree product representation arise from the reduction in the number of NEO-SCF iterations (Figure 3 in the main text), the per iteration evaluation is also slightly more efficient mainly because the nuclear exchange terms do not need to be computed.

| | Slater Determinant | | Hartree Product | |
|---|---|---|---|---|
| System | Iteration | Nuc J/K | Iteration | Nuc J |
| $H_3O^+$ | 0.14 | 0.08 | 0.06 | 0.00 |
| $H_5O_2{}^+$ | 0.17 | 0.09 | 0.09 | 0.01 |
| $H_9O_4{}^+$ | 0.31 | 0.10 | 0.23 | 0.03 |
| $H_{11}O_5{}^+$ | 0.49 | 0.10 | 0.39 | 0.04 |
| $H_{13}O_6{}^+$ | 0.59 | 0.11 | 0.53 | 0.06 |

# References

[1] D. B. Williams-Young, A. Petrone, S. Sun, T. F. Stetina, P. Lestrange, C. E. Hoyer, D. R. Nascimento, L. Koulias, A. Wildman, J. Kasper, J. J. Goings, F. Ding, A. E. DePrince III, E. F. Valeev, and X. Li, "The Chronus Quantum software package," Wiley Interdiscip. Rev. Comput. Mol. Sci. **10**, e1436 (2020).

[2] S. Lehtola and L. A. Burns, "OpenOrbitalOptimizer–A reusable open source library for self-consistent field calculations," J. Phys. Chem. A **129**, 5651–5664 (2025).
